# Challenges and opportunities for early childhood intervention services in Singapore: addressing user satisfaction

**DOI:** 10.3389/fpubh.2025.1566167

**Published:** 2025-07-01

**Authors:** Soojin Kim, Xinwei Zhang, Sachi Sawant, Kenneth K. Poon, Poh Choo Khoo, Chui Mae Wong, Lourdes Mary Daniel, Evelyn C. Law, Elene Lee, Victoria Leong

**Affiliations:** ^1^Public Policy and Global Affairs Programme, School of Social Sciences, Nanyang Technological University (NTU), Singapore, Singapore; ^2^Department of Psychology, School of Social Sciences, Nanyang Technological University (NTU), Singapore, Singapore; ^3^The Centre for Research in Child Development at the National Institution of Education, Nanyang Technological University (NTU), Singapore, Singapore; ^4^Department of Neonatology, KK Women's and Children's Hospital, Singapore, Singapore; ^5^Department of Child Development and Neonatology, KK Women's and Children's Hospital, Singapore, Singapore; ^6^Department of Child Development, KK Women's and Children's Hospital, Singapore, Singapore; ^7^Department of Paediatrics, Yong Loo Lin School of Medicine, National University of Singapore, Singapore, Singapore; ^8^Institute for Human Development and Potential, Agency for Science, Technology and Research, Singapore, Singapore; ^9^SingHealth, Singapore, Singapore; ^10^Early Mental Potential and Wellbeing Research (EMPOWER) Centre, Nanyang Technological University (NTU), Singapore, Singapore

**Keywords:** early childhood intervention, Singapore, COVID-19, multistakeholder satisfaction, policy

## Abstract

Since the COVID-19 pandemic, there has been a gap between primary stakeholders' *ex ante* expectations of adaptive early childhood intervention (ECI) services and their actual experiences with these services. This is despite governments' and service operators' swift pivot from on-site service delivery to home-based, virtual and hybrid modes, and cross-sector collaboration with private providers and pre-schools. In this article, we share our preliminary evidence from in-depth interviews with parents and clinicians regarding the perceived challenges to post-pandemic ECI services. We discuss how the crisis has triggered a paradigm change, especially regarding potential drivers for satisfactory services in the Singapore context.

## Introduction

Children's early years, particularly from birth to three, are often considered a sensitive period for neurodevelopment. This initial period from infancy through early childhood plays a tremendous role, shaping not only children's developmental trajectories but also their achievements in the later stage of life, such as their schooling outcomes, self-esteem, and family wellbeing (1–5). Accordingly, both scholars and practitioners have long emphasized the importance of effectively designing and delivering early childhood intervention (ECI) services for infants and young children at neurodevelopmental risk, including those with autism spectrum disorder (ASD), attention deficit hyperactivity disorder (ADHD), or who are born prematurely.

This article focuses on the ECI landscape in Singapore, a small, multicultural, and densely populated Southeast Asian nation. Unlike other countries such as the U.S., Singapore's ECI services take a broad age focus from 0 to 6 years, and also include early screening and detection within the scope of service definition. The number of preschoolers with developmental issues in Singapore has more than doubled from 2,502 in 2010 to 5,270 in 2019 (2, 6). Meanwhile, the average waiting time for enrolment in early intervention centers was ~7.5 months in 2023, an increase from 6 months in 2020 (7). To address these growing needs, the Singapore government has progressively expanded their collaborative partnerships with sectors beyond healthcare (8), such as social service agencies (SSAs), preschools, and government agencies including the Early Childhood Development Agency (ECDA) under the Ministry of Social and Family Development (MSF). Singapore's Early Intervention Programme for Infants and Children (EIPIC), launched in 2005, now provides comprehensive community-based ECI in services for children under 7 years of age. For children with mild needs, government agencies have focused on providing inclusive education in preschools with on-site educational support (i.e., Development Support and Learning Support [DS-LS], Development Support Plus [DS-Plus] and Inclusive Support Programme [InSP]). These programs create a more socially inclusive environment and allow children ample opportunities to interact with typically-developing peers (1, 9, 10, 35).

Given the growing demand for ECI services, this article explores underpinning challenges to the provision of adaptive ECI services since the COVID-19 pandemic, some of which are still on-going, through the eyes of parents and clinicians. In doing so, we hope to foster better preparedness for policymakers, educators, and developmental psychologists in the event of future public health emergencies that require a return to fully virtual services. Relatedly, we propose a new research agenda to develop a deeper understanding of primary stakeholders' satisfaction with the ECI services they are offered, taking into account their diverse needs.

## A lasting impact of the COVID-19 pandemic on early childhood services

In Singapore, as around the world, the COVID-19 pandemic and concurrent economic crisis disrupted ECI services and normal childhood activities (e.g., attending school, playing outdoors, and interacting with extended family and friends). Lockdown restrictions that required the temporary closure of educational centers stymied the smooth flow of essential ECI functions—for example, early detection and the creation of tailored goal- and skill-oriented treatment sessions (e.g., language/speech, emotional and behavioral regulation, motor and social skills), undermining the continuity, stability, and quality of ECI provision (11). Consequently, (working) parents experienced increased physical and emotional stress, juggling childcare, homeschooling, and job responsibilities within blurred work-family boundaries (12–16). Midway through the pandemic, sudden COVID-19 clusters led to repeated service suspensions for deep cleaning, which further burdened educators and clinicians already grappling with staffing shortages and burnout (17, 18).

To cope with these unprecedented challenges, governments and service providers swiftly replaced on-site screening and therapy sessions that would normally take place in Early Intervention (EI) centers/clinicians' offices with remote alternative care arrangements such as socially-distanced appointments, teleconsultations, and online home-based interventions. While these digital formats filled service gaps, limitations remained. Nearly 17.1% of caregivers felt consultations were incomplete without face-to-face assessments, and 88% of service providers reported difficulties, such as reduced observation, communication barriers, and poor connectivity (19).

Although the shift to digital delivery was rapid, the easing of restrictions lagged. Even now, more than 3 years after the pandemic's peak, there still remains an enduring gap between primary stakeholders' (here, parents and clinicians) *ex ante* expectations of ECI developmental services and their actual experiences, contributing to lower levels of satisfaction with service delivery.

## The evolution of ECI services based on “how-to” approaches

Supporting at-risk young children with developmental needs and social or behavioral disorders has been a longstanding area of scholarly debate, especially in the fields of psychology, medicine, education, and health policy. A considerable proportion of the literature on optimal ECI implementation has identified factors (conditions) that influence the effectiveness of ECI systems, mostly centered on institutional support and organizational capacity as critical enablers. For instance, most of the widely discussed facilitating factors include service quality, shorter waiting times, systematic and regular training for educators and professionals along with regularly updated hands-on training resources (1, 20–23), active and strong parent-professional communication (10), and enhanced public awareness of ECI's value in schools and communities (24).

In parallel to this research activity, there have also been noticeable advancements in ECI service delivery. These include the implementation of developmental surveillance (screening), diagnostic testing, and access to follow-up treatment opportunities through government-funded, community-based statewide programs, which rely on adequate societal support. In Singapore, as elsewhere, the intensity and quality of the educational and medical support system has advanced, and there has been a shift from *downward* management toward *upward* and *outward* management in a broader manner.

Historically, Singapore's ECI process began with conventional, diagnosis-focused interventions administered as early as possible in a child's life. These interventions were primarily led by healthcare professionals (clinicians or allied health specialists) at central assessment centers or publicly-designated hospitals (2, 25, 26). Over time, while the expectation has grown that substantial investment in ECI programs produces benefits at the family and community levels, this one-way, top-down style of intervention has progressively shifted toward a more holistic, family-centric approach, accommodating *bottom-up* interventions. The current model leverages the observations and assessments of multiple stakeholders including parents, clinicians (mostly trained pediatricians), preschool educators, caregivers, and social workers (27, 28). This transformation has also been accompanied by diversified government-led and government-aided programs and services, such as financial assistance schemes for economically-disadvantaged households (e.g., subsidized school fees or therapy sessions at the designated centers or public hospitals), course offerings (e.g., a training certificates or diplomas) or financial support for learning and skilling-building opportunities for educators and clinicians, and guidelines for parents and caregivers with at-risk children (e.g., standardized health booklets with developmental milestone checklists) (2). Together, these reforms reflect a shift toward adaptive, inclusive, and practical “how-to” approaches in ECI service delivery.

## Discrepancy between expectations and lived experiences since COVID-19

Despite commendable policy improvements and support schemes enabling families with limited knowledge or financial means to access high-quality ECI services, ensuring the satisfactory performance of ECI services—especially among primary service users and providers (here, parents and clinicians, respectively)—is no simple thing in practice. Following the recent public health crisis, there remain challenges in developing in-house capacity, establishing critical peripheral support for parent training and outreach, and even delivering remote services effectively.

Based on our in-depth analysis based on semi-structured interviews and focus group discussions,^1^ most participating parents cited “cost and time (scheduling) pressures” as the main barrier to their satisfaction with ECI services, saying:

“*I really want to see that both private and government subsidised, and big centres should consider having [services available] on weekends as well, because it's very difficult for us... because all the therapy is during working hours* … *We have weekend services at those that will be a little bit more costly*.” (Parent #13)

“*There's not sufficient infrastructure or vacancies to cater to people. So I feel that … we were sort of forced in a way, in some way, to go to private, because, you know, it was hard to get a public appointment, and the other challenge was the cost*..” (Parent #25)

“*Because he was born in the COVID-period, [we spent] a lot of time staying at home, got no place to go, so from there, we go for Zoom sessions, like once a week*.… *private therapy is very expensive. We are just waiting*.” (Parent #26)

Clinicians also commented along the same lines, for example:

“... *[There can be] quite a long wait time for early intervention programs, it can be 1 to 2 years. I think after COVID. So that is quite a lot of challenge...s That's why a lot of parents seek private [services]. I mean a hospital setting. We do have interim services for different therapy services but I think they're not that frequent of therapy service.… When it is more frequent it's also quite costly*.” (Clinician #19)

In addition, parents pointed out a “lack of clear guidelines or knowledge curation for parents (caregivers)” as another salient challenge, as illustrated in the following quotes:

“…* Currently there are many other services like the Early Intervention Programme for Infants & Children (EIPIC) and the development support (DS) programme for childcare, but nobody really explains to you what is good for your kids. What is the best option? Yeah. So, you really have to go find out yourself … in the information about the different kind of programs*.” (Parent #18)

“*I'm not really equipped with the resources and the knowledge. Okay, this is what you need to do if they are signed up, and they don't really encourage us to do that. So far, the polyclinic just told me to wait it out. … I think probably [that is] something that needs to be changed now*.” (Parent #23)

Related to this, they further indicated that “limited community-based resources” may inhibit the immediate seeking related information or the sharing of concerns across communities,

“*It would be best, it will make you feel relatable if I share my story with them, it means not only me, but lots of parents around me also go through this. I think there is a need, … to help them with coping and overcoming things like that*.” (Parent #13)

“*They can try to advocate the milestone... and try to encourage people to use community portals applications in your neighbourhood … we've more people facing this part, those who have a better understanding, especially parents, and therefore, perhaps they will allow special needs conditions to be detected earlier*.” (Parent #16)

“*I started following a lot of those social media influencers on Instagram and on Facebook instead. So, I copied some of their recipes but adapted it to suit him a bit more*.” (Parent #28)

Clinicians expressed similar views, noting the scarcity of professional resources and the potential harm of unverified social media content, often a mix of fragmented or misleading information:

“… *For a parent who's new to the setting, it may be difficult to navigate these community resources. That you know. How do I access them? What is good, what is not good? What fits my needs?*” (Clinician #9)

“*There are things not publicised more … Sometimes I don't even know about this website. And then, unless you really dwell, like spend a lot of time on this website to find out what you really need*.... *think about how we can maybe [find] good resources. If you have too many resources out there on the World Wide Web, it's so hard for parents to surf the web, you might want to, you know, just come together to narrow down*.” (Clinician #25)

“… *These days there are lots of news out there. So, our parents are flooded with information, and some of it is misinformation. But they're not very sure; they may not know how to differentiate*.” (Clinician #26)

The findings of our qualitative study should be considered preliminary, but their implications are not far-fetched in the context of understanding the post-pandemic needs of the ECI sector. Parents' anxiety often stemmed from limited access to timely, curated resources, weak organizational structure, and insufficient two-way communication between stakeholders. Our study similarly identifies key barriers to satisfaction with ECI services as in previous research [e.g., (20, 21, 23)], but it extends previous literature by highlighting parents' need for flexible service delivery (e.g., subsidized weekend sessions), and capturing clinicians' concerns. Interestingly, the clinicians largely echoed these concerns, though they also expressed caution about parents' heavy reliance on unverified online content. Most challenges were identified in the implementation phase of treatment—when children begin intervention or transition to preschools—rather than during the initial phase of detection and identification of special needs.

The abovementioned evidence hints at some policy innovations that deserve further discussion and investment. For example, these include expanding partnerships with more SSA/private-sector EI operators, supported by government grants to enhance affordability (especially to financially-vulnerable families), to offer more flexible and subsidized session schedules. To bridge communication and information gaps, appointing dedicated liaison officers or leveraging specialized organizations (such as SGEnable) as intermediaries between families, educators, and healthcare providers could improve coordination among stakeholders—especially between parents and clinicians or school educators. Additionally, diversifying official communication channels and strengthening the curation of user-friendly, evidence-based resources via community-based or accessible digital platforms would help parents and caregivers become better aware of and navigate the available information. These platforms can also provide a space for parents to share their concerns and feel heard, fostering greater empathy.

## Discussion: lessons learned from challenges and the path toward best practices

Evidence gleaned from our interviews shows that the value of current ECI-related services, especially the degree to which existing public programs and services meet and reflect the primary stakeholders' diverse needs and demands, may be underestimated in part due to *information asymmetries* between policy designers (here, public agencies) and policy beneficiaries (here, parents), and between service users (here, parents) and service providers (here, clinicians). To better understand this, it may be instructive to take a deeper dive into the service user's basis for evaluation.

It has been long argued that because citizens are the main service users who actually consume a given public service, their opinions can effectively mirror and complement government-led objective assessments or other quantitative assessments of organizational performance (29–31). One of the most widely cited theoretical frameworks in the public administration and policy literature is the expectation-disconfirmation model (EDM), which posits that an individual evaluates service performance against a particular reference point (prior expectations). A mismatch between the two generates a so-called “disconfirmation” effect, which can be positive or negative (29, 32–34). The effect occurs via the interplay of individuals' *ex ante* expectations and their *ex post* satisfaction based on their lived experiences. For example, when citizens hold high expectations and the service fails to meet them, negative disconfirmation occurs, leading to lower levels of satisfaction (dissatisfaction).

Following this line of reasoning and expanding the theoretical lens to other actors, we propose a new line of inquiry: “*How do service users (policy recipients) and providers (policy operators) perceive whether ECI services and policies meet their intended goals, as reflected in their satisfaction with the process and outcomes?*” It can reasonably be expected that developmental scientists, policymakers, and local authorities would benefit from an examination of the effectiveness of ECI programs/services from this angle. By incorporating the voices of parents and clinicians, it offers a chance to close the policy feedback loop and promote greater diversity, equity, and fairness. Given that the transition from conventional in-person care via hospital/center visits toward a hybrid mode that encompasses on-site therapy sessions and online interventions has been readily apparent since the COVID-19 pandemic (13, 16), we are optimistic that providers can realize new benefits from this opportunity to further adapt their strategies to improve ECI services in more family-centered and reassuring ways.

Furthermore, many existing studies on ECI services have lacked particular scrutiny of perceived service performance or future policy directions for the overall ECI landscape. Quantitative analyses based on survey-driven data or large-scale numerical datasets still predominate, which may hinder a more nuanced understanding of the issue. Hence, we argue that the methods of inquiry must be broadened and sharpened in order to better understand the factors (conditions) that contribute to effective and satisfactory ECI service delivery. We strongly encourage scholars and practitioners to identify best practices via methodologically rigorous investigations that combine observations, interviews, focus group discussions, and survey questionnaires. These can enrich the existing literature, not only in the areas of psychology, medicine, education, and sociology, but also in adjoining fields that address contemporary social and health policy issues, such as public administration and policy. Such approaches can help uncover practical strategies that are often overlooked in the existing institutional, organizational, and relational contexts.

ECI services are vital and transformative for children, families, and communities. Strengthening ECI-related programs and services can be considered a “wicked” problem that requires a combination of socio-economic, institutional, environmental, and cultural support and attention. In this vein, we posit that one of the logical next steps for future research is to diagnose and monitor the effectiveness of currently offered ECI services through the eyes of various stakeholders. By doing so, we can identify the acceptable benchmarks or preconditions of stakeholders on both the demand and supply sides, which influence their level of satisfaction with the provided services. Further, it will foster a more comprehensive understanding of what constitutes successful long-term interventions that support early childhood development.

## Data Availability

The original contributions presented in the study are included in the article/supplementary material, further inquiries can be directed to the corresponding author.

## Footnotes

^1^Interviewees were recruited through clinician referrals and promotional materials (e.g., posters and pamphlets) distributed at outpatient clinics of KK Women's and Children's Hospital, as well as through social media platforms of Nanyang Technological University, Singapore. A total of 60 in-depth interviews (33 parents and 27 clinicians) were conducted virtually (e.g., via Zoom) between April 2023 and November 2024.

## References

[1] Ang L Lipponen L May Yin SL. Critical reflections of early childhood care and education in Singapore to build an inclusive society. Policy Futures Educ. (2021) 19:139–54. 10.1177/1478210320971103

[2] Ho LY. Current status of the early childhood developmental intervention ecosystem in Singapore. Singapore Med J. (2021) 62:S43–52. 10.11622/smedj.2021076

[3] Okoye C Obialo-Ibeawuchi CM Obajeun OA Sarwar S Tawfik C Waleed MS. Early diagnosis of autism spectrum disorder: a review and analysis of the risks and benefits. Cureus. (2023) 15:e43226. 10.7759/cureus.43226 37692637 PMC10491411

[4] Poon K Xie H Yang X. Early childhood intervention for young children with special needs in singapore: where we have been and future directions. In: Early Childhood Development and Education in Singapore. Singapore: Springer (2022). p. 99–112.

[5] Xie H Nah YH Yang X Sengalrayan BW Poon KK. Early childhood intervention: what we know and where we are headed. A review of local and international literature and implications for Singapore. In: NIE Working Paper Series No. 18. Singapore: National Institute of Education (2021).

[6] Statista. Total Population of Singapore from 1990 to 2023 (in millions). (2024). Available online at: https://www.statista.com/statistics/713063/singapore-total-population/ (accessed February 21, 2025).

[7] Li TW Ang S. Long wait for subsidised support pushes parents of kids with developmental needs to private sector. The Straits Times. (2024). Available online at: https://www.straitstimes.com/singapore/long-wait-times-for-early-intervention-push-parents-of-kids-with-developmental-needs-to-private-sector

[8] Kim S Goh Y Kang JHB. Moving toward a common goal via cross-sector collaboration: Lessons learned from SARS to COVID-19 in Singapore. Global Health. (2022) 18:82. 10.1186/s12992-022-00873-x 36131348 PMC9490717

[9] Early Childhood Development Agency (ECDA). Advancing Support for Children in Preschools - Greater Accessibility, Affordability, Quality and Inclusion. (2021). Available online at: https://www.ecda.gov.sg/news/press-releases/advancing-support-for-children-in-preschools-greater-accessibility-affordability-quality-and-inclusion (March 5, 2021).

[10] Xie H Poon KK. Maximizing participation in preschool education of young children with developmental delays/disabilities in Singapore: building a continuum of early childhood intervention service delivery. In:Acar S Hix-Small H McLaughlin T, editors. Young Exceptional Children Monograph Series No. 18: International Perspectives on Early Intervention, Early Childhood Special Education (EI/ECSE) Longmont, CO: Sopris West (2019). p. 24–32.

[11] Davies C Kong SP Hendry A Archer N McGillion M Gonzalez-Gomez N. Sustained benefits of early childhood education and care (ECEC) for young children's development during COVID-19. J. Early Childhood Res. (2024) 22:238–57. 10.1177/1476718X231213488

[12] Borg A Dulk LD Lewis S Santos C. Community, work and family in diverse contexts and changing times. Community Work Family. (2020) 23:497–502. 10.1080/13668803.2020.1832264

[13] Kwok J Winston S Gerdes M Morales K McQuaid E Guevara JP. Impact of the COVID-19 pandemic on early intervention services use among children with developmental disabilities. J Pediatr Adv Res. (2024) 3:1–9. 10.46889/JPAR.2024.3101 40213490 PMC11984540

[14] Madayi A Padmini YS Wong CM. The impact of Singapore's COVID-19 circuit breaker measures on children with developmental delays and their families. Asia Pacific J Pediatr Child Health. (2022) 5:A211–A212. 10.1136/archdischild-2021-rcpch.369

[15] Vilaseca R Ferrer F Rivero M Bersabé RM. Early intervention services during the COVID-19 pandemic in Spain: toward a model of family-centered practices. Front Psychol. (2021) 12:738463. 10.3389/fpsyg.2021.738463 34858273 PMC8631765

[16] Yang X Wong ME Poon KK. Emergency remote learning for children with disabilities during the pandemic: Navigating parental roles and supports. J Child Fam Stud. (2024) 33:439–57. 10.1007/s10826-023-02760-4

[17] Fonsén E Marchant S Ruohola V. Leadership in early childhood education: Cross-cultural case studies before and during the COVID-19 pandemic. Educ Manage Administr Leadersh. (2023). 10.1177/17411432231194849

[18] Qing A Patchymuthu L. Pre-Schools Grappling with COVID-19 Rules as More Children, Staff Infected. (2022). Available online at: https://www.straitstimes.com/singapore/parenting-education/pre-schools-grappling-with-covid-19-protocols-as-more-children-staff-infected (accessed February 20, 2022).

[19] Tan MY Kang YQ Kiing JSH Chong SC Mulay KV Lim TSH. Use of telemedicine in a developmental and behavioural paediatric service during the COVID-19 pandemic: Initial experiences of caregivers and providers in Singapore. Singapore Med J. (2023). 10.4103/singaporemedj.SMJ-2021-003 PMC13143316 41885258

[20] Compania DV Caberte J Guarin M Jerios M Ablando NG. Non- SPED Teachers' Readiness for Inclusive Education. Excellencia: International Multi-Disciplinary Journal of Education (2994-9521). (2024) 2:354–63. 10.5281/zenodo.10608311

[21] Lloyd E Penn H. Childcare Markets: Can They Deliver an Equitable Service? UK: Policy Press (2012). 10.1332/policypress/9781847429339.001.0001

[22] Odom SL Buysse V Soukakou E. Inclusion for young children with disabilities: a quarter century of research perspectives. J Early Interv. (2011) 33:344–56. 10.1177/1053815111430094

[23] Tan C. Private supplementary tutoring and parentocracy in Singapore. Interchange. (2017) 48:15–329. 10.1007/s10780-017-9303-4

[24] Poon KK Yang X. The student profile, service delivery model, and support practices of four early childhood intervention environments in Singapore. Asia Pacific J Educ. (2014) 36:437–49. 10.1080/02188791.2014.940030

[25] Ho LY. Childhood development programme in Singapore 1988 to 2007. Ann Acad Med. (2007) 36:898–910. 10.47102/annals-acadmedsg.V36N11p898 18071596

[26] Quah MM. Family-centred early intervention in Singapore. Int J Disabil Dev Educ. (1997) 44:53–65. 10.1080/0156655970440105

[27] Chong WH Goh W Tang HN Chan WP Choo S. Service practice evaluation of the early intervention programs for infants and young children in Singapore. Children's Health Care. (2012) 41:281–301. 10.1080/02739615.2012.721719 21668465

[28] Poon KK Lim A-K. Current provision, recent developments, and future directions for early childhood intervention in Singapore. Infants Young Child. (2012) 25:323–33. 10.1097/IYC.0b013e31826615f9

[29] Kim S Rho E Teo YXJ. Citizen satisfaction research in public administration: A systematic literature review and future research agenda. Am Rev Public Administr. (2024) 54:460–85. 10.1177/02750740241237477

[30] Kim S Kim M. Citizen satisfaction in the public sector. In Schedler K., editor. Elgar Encyclopedia of Public Management. Cheltenham: Edward Elgar Publishing Limited (2022). p. 205–7.

[31] Lee JB Kim S. Understanding gaps between objective and subjective performance measures: accreditation of public service organizations and citizen satisfaction. Am Rev Public Administr. (2024) 54:271–86. 10.1177/02750740231193431

[32] James O. Managing citizens' expectations of public service performance: evidence from observation and experimentation in local government. Public Adm. (2011) 89:1419–35. 10.1111/j.1467-9299.2011.01962.x

[33] Oliver RL. A cognitive model of the antecedents and consequences of satisfaction decisions. J Market Res. (1980) 17:460–9. 10.1177/00222437800170040523326940

[34] Van Ryzin GG. Testing the expectancy disconfirmation model of citizen satisfaction with local government. J Public Administr Res Theory. (2006) 16:599–611. 10.1093/jopart/mui058

[35] Early Childhood Development Agency (ECDA). FAQs – Early Intervention Programme for Infants and Children – P (EIPIC-P). (2024). Available online at: https://www.enablingguide.sg/docs/default-source/default-document-library/faqs-eipic-p.pdf?sfvrsn=30e7e8f4_12 (accessed January 12, 2024).

